# Time utilities: programs in MUMPS for calculating intervals of time and for timing events

**DOI:** 10.1155/S1463924686000391

**Published:** 1986

**Authors:** T. G. Pellar, A. R. Henderson

**Affiliations:** Department of Clinical Biochemistry University Hospital (University of Western Ontario) P.O. Box 5339 Postal Stn. A London Ontario N6A 5A5 Canada


Journal of Automatic Chemistry/*Journal of Clinical Laboratory Automation*, Vol. 8, No. 4 (October–December 1986), pp. 202–206

### Time utilities: programs in MUMPS for calculating intervals of time and for timing events

**T. G. Pellar and A. R. Henderson**

*Department of Clinical Biochemistry, University Hospital (University of Western Ontario), P.O. Box 5339, Postal Stn. A, London, Ontario, Canada N6A 5A5 Canada N6A 5A5*

### Introduction

The MUMPS language possesses a special function, $HOROLOG (or $H), which allows easy manipulation of dates and times [1 and 2]. $HOROLOG returns a string containing two numbers, D and S. The day counter, D, starts in standard MUMPS from the first day of January 1841; the second counter, S, starts afresh at midnight on each day and counts elapsed time in seconds.

There are several routines available to calculate time from the S counter and the date from the D counter [1 and 2]; two are provided in the Appendix. Times may be expressed in 24-hour clock notation (for example 1850 h) or in a.m./p.m. (for example 6.50 p.m.) style. Dates can be expressed in a variety of formats to suit local requirements (or prejudices) such as 2 Jan 1985, Jan 2 1985, 020185, 02/01/85, 010285, 850102 and so on. Clearly this wide flexibility in final style, the availability of the day and second counters and the general ease of use of the MUMPS language anyway makes it very easy to create programs for solving problems involving dates or times.

A set of simple programs are described in this paper; the programs are used daily, in a hospital department of clinical biochemistry, for event timing and calculations involving intervals and days, or hours, of patient stay.

### Materials and methods

The University Hospital laboratory computer system is a 32-bit Data General Eclipse MV/6000 with 1 megabyte of core memory (Data General [Canada] Ltd, Mississauga, Ontario, Canada). The XL-87H video display terminal (Cybernex Ltd, Ottawa, Ontario, Canada) was used; this emulates a Hazeltine terminal. The computer operating and programming system was written in the MIIS dialect of MUMPS (Medical Information Technology, Inc., Cambridge, Massachusetts, USA) [3]. An explanation of some of the MIIS-specific functions used in the programs is provided in the Appendix.

The operating system allows direct access to the MUMPS programming mode by means of passwords. Operating in this direct mode does not interfere with the main laboratory system or its data-base.

### The program

On calling the time utilities, the operator is asked to select from:

Elapsed Time Calculator
Set Time Calculator
TS-Time to a Specific Time
TL-Time to a Specific Length of Time.

These are selected by entering the first or first and second letters of the required option. The program for this menu is listed in table 1.

*Table 1. The menu for the time utilities. This is displayed to the operator after signing on.*

```

TIME# ;MENU FOR TIME UTILITIES 140286 TGP

1      W #!?33,"TIME UTILITIES",! F I=1:1:19 W "//\\"

       G:$DEF(IN) 3

2      A 0:N=1 R !,"Your code: ",CODE A 0:N=""

       I '$D(^TECHC(CODE)) Q

       S BF="BAD FORMAT",IN=^TECH(^TECHC(CODE))N,R=^TECHC(CODE) W IN[1]:3

       W !!,"Elapsed Time Calculator",!,"Set Time Calculator",!,"TS-Time to a Spe

          cific Time",!,"TL-Time for a Specific Length of Time"

3      R !!,"Option: ",A Q:'A W:A="?" ! S B

4      S B=B+1,C=$T(5+B) I 'C W:A'="?" *7,"??":3 G 3

       I A="?" W !,$P(C;2) G 4

       I $E($P(C;2),1,$L(A))=A W $E($P(C;2),$L(A)+1,99),! H 1 X $P(C;3,99) G 3

       G 4

5      ;LIST OF TIME OPTIONS

       ;Elapsed Time Calculator;C TPET1

       ;Set Time Calculator;C TPTIME2

       ;TS-Time to a Specific Time;C TPET4@TIME

       ;TL-Time for a Specific Length of Time;C TPET4

       G 4

```

### The elapsed time calculator

This routine calculates the elapsed time from the initial event to any subsequent event(s). The opening sequence is:

Time (24 h clock): 1321

Date format is DD MM YY, current date is: 26 Ø3 86.

This establishes the format to be used. The operator is then asked:

Initial event (time=Ø):

This can either be answered with a date (for example 23 Ø1 86) or with both time and date (for example 1213 13 Ø3 86). The response is:

Subsequent event:

Again this can be answered with another date (for example 25 Ø3 86) or with a time and date (for example Ø344 14 Ø3 86). In the former case the response is:

Subsequent event: 25 Ø3 86

Elapsed time : 61 d since 23 Ø1 86

and in the latter case:

Subsequent event : Ø344 14 Ø3 86

Elapsed time: 15 h 31 min (15.52 h) since 1213 13 Ø3 86.

It is a tedious and error-prone step to enter the entire time and date for each subsequent event. A series of defaults has therefore been inserted in the program that will allow entry only of the new elements of the date. Thus, if only the time is different from the initial event it is only necessary to press <ENTER> after entry of the time. If both the time and the day are different, then after these have been entered the program will use the previous (i.e. initial event) month and year setting. This default has proved to be extremely useful. Thus when timing samples from the onset of chest pain taken from a patient with a myocardial infarction [4], four or five specimens have often been found with the same date so that this default considerably reduces the amount of data to be keyed in. This routine is most commonly used when all patient data is available. The program is listed in table 2. When patients are being studied over longer periods, with intermittent entry of data, the next routine to be described is more suitable.

### Set time calculator

This routine performs the same function as the Elapsed Time Calculator except that a file is opened for each patient, together with the date and time of the initial event. This type of routine is useful when following a patient, or experimental subject, over weeks, months or even years. For example, in a study of serum enzyme changes in heart transplant patients [5], some patients were followed for over two years.

The routine has five components – Input, Edit, Delete, List and Calculate. Input is used to open a file:

Title for Set Time: Toby Belch
Set Time (format HHMM DD MM YY): 1Ø51 27 Ø3 86//.

The file title is entered and the Set Time established (present time and date are shown as an example of the format). If any subsequent time is now to be entered call Calculate:

Which Set Time: ? (entering a '?' to list the files)

| Set Time # | Name | Set Time |
|---|---|---|
| (1) | Toby Belch | 1531 Ø6 Ø1 85 |
| (2) | Andrew Aguecheek | Ø2Ø5 17 Ø6 85 |
| (3) | Roderick Random | 12Ø1 25 11 85 |
| | and so on | |

Which Set Time: (enter the Set Time #) 1 <ENTER>

(response) 1 Toby Belch 1531 Ø6 Ø1 85

Calculate time to: 14ØØ Ø9 Ø1 85

Elapsed time:1 d 22 h 29 min (70.48 h)

Calculate time to: and so on.

Alternatively, only the date may be entered for initial time. Thereafter the routine will only accept entries in date form. Elapsed time is calculated in days. This calculation is valuable when calculating days of hospital stay, or of survival. This program segment is listed in table 3.

### Time to a specific time

This routine allows the video terminal to be used as a clock. On calling the option the user is given the current time and asked what time the alarm is to ring:

Present time: 1Ø:23:5Ø

Time to ring alarm [Format hh:mm:ss or hh:mm]: 1Ø:35

(Press enter to start timer) <ENTER>

The screen is cleared and then displays:

Alarm time: 10:35
Present time. 10:25:42
(present time refreshes every second).

When 'present time' reaches 'alarm time' the terminal will emit a beep, and the system returns to the Options Menu. This program segment is listed in table 4.

### Time for a specific length of time

This routine allows the video terminal to be used as an interval timer. The procedure is as follows:

Inverval to time for:-
hours: minutes: Seconds:

(Press enter to start timing) <ENTER>.

The screen is cleared and then displays:

Time remaining 00:01:59
(display refreshes every second)

When time remaining is zero, the terminal will emit a beep and the system returns to the Options Menu. The program segment is listed in table 4.

*Table 2. The program for the Elapsed Time calculation. This program will accept dates alone (output then in days) or dates and times (output in days and hours) for interval calculations.*

```

TPET1 .ELAPSED TIME CALCULATOR 201285 TGP

      K (IN,R,BF) D $DT[$T/86400+64223,"DT","PPNO"] S DD=$E(DT,1,2),MD=$E(DT,3,4

        ),YD=$E(DT,5,6)

      S HP=$T#86400/3600 I $L(HP)=1 S HP=0_HP

      S MP=$T#86400#3600/60 I $L(MP)=1 S MP=0_MP

      W !,"Time(24 h clock): ",HP,MP,"Date format is DD MM YY, current date is:

        ":4,DD:1,MD:1,YD:1

1     R !!,"Initial event (time=0): ",INT I 'INT K (IN,R,BF) Q

      I $L($P(INT 1))=4 D 2

      E  C TPET2

      G 1

2      CALCULATE IN SEC

      S TI=$P(INT 1),DI=$P(INT 2),MI=$P(INT 3),YI=$P(INT 4)

      I TI'="2400" I $E(TI,1,2)="24" S TI=9999

      I 'DI S DI=DD W DI:1

      I $L(DI)=1 S DI=0_DI

      I 'MI S MI=MD W MI:1

      I $L(MI)=1 S MI=0_MI

      I 'YI S YI=YD W YI:1

      I (($L(TI)'=4)!($L(DI)'=2)!($L(MI)'=2)!($L(YI)'=2)) W *7,BF:3 Q

      S PT=TI,PD=DI,PM=MI,PY=YI

      S DATI=DI_MI_YI

      D $DR[DATI,"DAYI"] I 'DAYI W *7,BF:3 Q

      C %TR@I[TI,"TIS"] I 'TIS W *7,BF:3 Q

      S SECI=DAYI*86400,SECI=SECI+TIS I TI="2400" S SECI=SECI+86400 W !

3     ;READ IN D&T'S FOR ELAPSED TIME

      S FL R !!,"Subsequent event: ",ET I 'ET K (IN,R,BF,DD,MD,YD) Q

      S TE=$P(ET 1),DE=$P(ET 2),ME=$P(ET 3),YE=$P(ET 4)

      I TE'="2400" I $E(TE,1,2)="24" S TE=9999

      I 'DE S DE=DI W DE:1

      I $L(DE)=1 S DE=0_DE

      I 'ME S ME=MI W ME:1

      I $L(ME)=1 S ME=0_ME

      I 'YE S YE=YI W YE:1

      I (($L(TE)'=4)!($L(DE)'=2)!($L(ME)'=2)!($L(YE)'=2)) W *7,BF:3 G 3

      S DATE=DE_ME_YE

      D $DR[DATE,"DAYE"] I 'DAYE W *7,BF:3 G 3

      C %TR@I[TE,"TES"] I 'TES W *7,BF:3 G 3

      I DE'=DI S DI=DE

      I ME'=MI S MI=ME

4     I YE'=YI S YI=YE

      S SECE=DAYE*86400,SECE=SECE+TES I TE="2400" S SECE=SECE+86400

      S ELS=SECE-SECI I ELS<0 S FL="-",ELS=ELS*-1

      S DO=ELS/86400 S YO=DO/365 I 'YO S YO=0

      S DO=DO#365 I 'DO S DO=0

      S HO=ELS#86400/3600 I 'HO S HO=0

      S MO=ELS#86400#3600/60 I 'MO S MO=0

      S DH=$M(ELS/3600+.005,.2)

      W !,"Elapsed time: " W:FL FL,"[ " W:YO'=0 YO," y  "

      W:((YO'=0)&(DO=0))!(DO'=0) DO," d  "

      W:(((YO'=0)!(DO'=0))&(HO=0))!(HO'=0) HO," h  "

      W MO," min","( ",DH,"h"," )" W:FL "]" W "since":3,PT:1,PD:1,PM:1,PY:1 G 3

TPET2 ;PART 2 ELAPSED TIME CALCULATOR 090186 TGP

      S DI=$P(INT 1),MI=$P(INT 2),YI=$P(INT 3)

      I $L(DI)=1 S DI=0_DI

      I 'MI S MI=MD W MI:1

      I $L(MI)=1 S MI=0_MI

      I 'YI S YI=YD W YI:1

      I (($L(DI)'=2)!($L(MI)'=2)!($L(YI)'=2)) W *7,BF:3 Q

      S PD=DI,PM=MI,PY=YI

      S DATI=DI_MI_YI

      D $DR[DATI,"DAYI"] I 'DAYI W *7,BF:3 Q

1     ,READ IN ELAPSED DAYS

      K FL R !!,"Subsequent event: ",ET I 'ET K (IN,R,BF,DD,MD,YD) Q

      S DE=$P(ET 1),ME=$P(ET 2),YE=$P(ET 3)

      I 'ME S ME=MI W ME:1

      I $L(ME)=1 S ME=0_ME

      I 'YE S YE=YI W YE:1

      I (($L(DE)'=2)!($L(ME)'=2)!($L(YE)'=2)) W *7,BF:3 G 1

      S DATE=DE_ME_YE

      D $DR[DATE,"DAYE"] I 'DAYE W *7,BF:3 G 1

      I ME'=MI S MI=ME

      I YE'=YI S YI=YE

      S ELD=DAYE-DAYI I ELD<0 S FL="-",ELD=ELD*-1

      S ELY=ELD/365 I 'ELY S ELY=0

      S ED=ELD#365

      W !,"Elapsed time: " W:FL FL,"[ " W:ELY'=0 ELY," y  " W ED," d " W:FL "]"

        W "since":1,PD:1,PM:1,PY:1 G 1

```

*Table 3. The program for the Set Time routine. This program performs the same functions as the Elapsed Time program but allows for the creation of a file for each subject so that calculations can be made at intervals during a study.*

```

TPTIME2 ;MENU FOR SET TIME 210386 TGP

1       R !!,"Set Time option: ",A Q:'A W:A="?" !! S B

2       S B=B+1,C=$T(3+B) I 'C W:A'="?" *7,"??":3 G 1

        I A="?" W !,$P(C;2) G 2

        I $E($P(C;2),1,$L(A))=A W $E($P(C;2),$L(A)+1,99),! H 1 X $P(C;3,99) G 1

        G 2

3       ;LIST OF TIME OPTIONS

        ;Input;C TPTIME4

        ;Edit;C TPTIME4@EDIT

        ;Delete;C TPTIME4@DEL

        ;List;C TPTIME5

        ;Calculate;C TPTIME4@INT

        G 4

TPTIME4 ;SET TIME ROUTINES 240386 TGP

        R !,"Title for Set Time:  ",TT I 'TT K (IN,R,BF) Q

        S SI=$T+5548867200 C TPTIME5@NOW S T=HN_MIN_" "_DN_" "_MN_" "_YN C TPTIME5

          @CON

        W !,"Set time (format HHMM DD MM YY): ",HN,MIN,DN:1,MN:1,YN:1," // " R T I

           T C TPTIME5@CON I 'SI W BF:3 Q

        S U=$U(^TPTIME(R))

        S ^TPTIME(R,U)[1]=TT,^TPTIME(R,U)[2]=SI,^TPTIME(R,U)[3]=FL

        Q

EDIT    ;EDIT TIME

        R !,"Which Set Time (by number): ",U I 'U K (IN,R,BF) Q

        I U="?" W ! C TPTIME5 G EDIT

        I '$D(^TPTIME(R,U)) W *7,"??":3 G EDIT

        W !!,"Title for Set Time: " W ^TPTIME(R,U)[1]," // " R X I X S ^TPTIME(R,U

          )[1]=X

        W !,"Set time: " S SI=^TPTIME(R,U)[2] C TPTIME5@NOW W HN,MIN,DN:1,MN:1,YN:

          1," // " R T I T C TPTIME5@CON I SI S ^TPTIME(R,U)[2]=SI,^TPTIME(R,U)[3

          ]=FL

        W ! G EDIT

DEL     ;DELETE TIME

        R !,"Which Set Time (by number): ",U I 'U K (IN,R,BF) Q

        I U="?" W ! C TPTIME5 G DEL

        I '$D(^TPTIME(R,U)) W *7,"??":3 G DEL

        W !,"OK to delete: ",^TPTIME(R,U)[1],"? N // " R ANS

        I $E(ANS,1,1)="Y" K ^TPTIME(R,U) Q

        Q

INT     ;CALCULATE

        R !!,"Which Set Time (by number): ",U I 'U K (IN,R,BF) Q

        I U="?" W ! C TPTIME5 G INT

        I '$D(^TPTIME(R,U)) W *7,"??":3 G INT

        W ^TPTIME(R,U)[1]:3 S SI=^TPTIME(R,U)[2],FLI= TPTIME(R,U)[3],FL=FLI C TPTI

          ME5@NOW W HN:2,MIN,DN:1,MN:1,YN:1 S SEC=SI

IN      R !!,"Calculate time to: ",T G:'T INT

        C TPTIME5@CON I 'SI G IN

        S FLS=FL I FLI'=FLS W:FLI=1 !,"Set time was input using time and date; you

          r input must use time and date." W:FLI=2 !,"Set time was input using on

          ly date; your input must use only date." W *7 G IN

        S EL=SI-SEC,FS I EL<0 S FS="-",EL=EL*-1

        S DO=EL/86400 S YO=DO/365 I 'YO S YO=0

        S DO=DO#365 I 'DO S DO=0

        S HO=EL#86400/3600 I 'HO S HO=0

        S MO=EL#86400#3600/60 I 'MO S MO=0

        S DH=$M(EL/3600+.005,.2)

        W !,"Elapsed time: " W:FS FS,"[ " W:YO'=0 YO," y  "

        W:((YO'=0)&(DO=0))!(DO'=0) DO," d  "

        I FL=1 W:(((YO'=0)!(DO'=0))&(HO=0))!(HO'=0) HO," h  "

        I FL=1 W MO," min","( ",DH,"h"," )"

        W:FS "]" G IN

TPTIME5 ;SET TIME UTILITIES 240386 TGP

        W !!?7,"Set Time #",?26,"Name",?55,"Set Time"

        W !! S U

DIS     S U=$N(^TPTIME(R,U)) I 'U W ! Q

        W !?8,U:1R3,")",?23,^TPTIME(R,U)[1]

        S SI=^TPTIME(R,U)[2],FL=^TPTIME(R,U)[3]

        D NOW W ?53,HN,MIN,DN:1,MN:1,YN:1

        G DIS

CON     ;CONVERT INPUT TO SECONDS

        S SI,TS,DAYI,TI

        I T'?4N" "2N" "2N" "2N I T'?2N" "2N" "2N W "??":1,*7 Q

        I $L($P(T 1))=4 S TI=$P(T 1),DI=$P(T 2),MI=$P(T 3),YI=$P(T 4),DATI=DI_MI_Y

          I,FL=1

        E  S DI=$P(T 1),MI=$P(T 2),YI=$P(T 3),DATI=DI_MI_YI,FL=2

        D $DR[DATI,"DAYI"] I 'DAYI W BF:3,*7 Q

        I TI C %TR@I[TI,"TS"] I 'TS W BF:3,*7 Q

        S SI=DAYI*86400,SI=SI+TS I TI="2400" S SI=SI+86400

        Q

NOW     D $DT[SI/86400,"DT","PPNO"]

        S DN=$E(DT,1,2),MN=$E(DT,3,4),YN=$E(DT,5,6)

        S HN=SI#86400/3600 I $L(HN)=1 S HN=0_HN

        S MIN=SI#86400#3600/60 I $L(MIN)=1 S MIN=0_MIN

        I (FL=2)!(FLI=2) K HN,MIN

        Q

```

*Table 4. The programs for the two timer utilities – Time to a Specific Time and Time for a Specific Length of Time.*

```

TPET4 ;TIMER FOR A SPECIFIC INTERVAL 140185 TGP

      W !,"Interval to time for: "

      R ?$X+2,"hours: ",TH W:'TH 0 R ?$X+2,"minutes: ",TM W:'TM 0 R ?$X+2,"secon

        ds: ",TS W:'TS 0

      S THS=TH*3600,TMS=TM*60,TT=THS+TMS+TS

      R !!!,"<Press enter to start timing>",X

      S TF=$T+TT

1     R TX:1/99999 Q:TX S TP=TF-$T

      S HL=TP/3600 I 'HL S HL=00

      I $L(HL)=1 S HL=0_HL

      S ML=TP#3600/60 I 'ML S ML=00

      I $L(ML)=1 S ML=0_ML

      S SL=TP#60 I 'SL S SL=00

      I $L(SL)=1 S SL=0_SL

      S TPP=HL_":"_ML_":"_SL

      W:TP#1=0 #!!!!!!!!!!!!?28,"Time remaining: ",TPP I (TF=$T)!(TF<$T) W *7 K (

        IN,R,BF) Q

2     I TF-$T'=TP G 1

      E  G 2

TIME  ;STOP AT A SPECIFIC TIME

      R !,"Time to ring alarm <Format hh:mm:ss or hh:mm>: ",TH Q:'TH

      I TH'?2N":"2N":"2N I TH'?2N":"2N W "FORMAT IS 'HH:MM:SS' OR 'HH:MM'":3 G T

        IME

      S SH=$P(TH:1),SM=$P(TH:2),SS=$P(TH:3)

      I SH>23 W *7,BF:3 G TIME

      I SM>59 W *7,BF:3 G TIME

      I SS>59 W *7,BF:3 G TIME

      S TI=SH_SM

      C %TR@I[TI,"TIS"] I 'TIS W *7,BF:3 G TIME

      D $DR["T","DAY"] S DE=(DAY-64223)*86400

      S DS=DE+TIS+SS

      R !!!,"< Press enter to start timer >",X Q:X

A     R X:1 Q:X'=""

      S HP=$T#86400/3600 I $L(HP)=1 S HP=0_HP

      S MP=$T#86400#3600/60 I $L(MP)=1 S MP=0_MP

      S SP=$T#60 I $L(SP)=1 S SP=0_SP

      W #!!!!!!!!!!!!!?30,"Alarm time: ",TH,!!?28,"Present time: ",HP_":"_MP_":"_

        SP

      I (DS=$T)!(DS<$T) W *7 K (IN,R,BF) Q

      G A

```

### Discussion

Calculations involving times or dates are common in medical and biological research. However, these calculations are awkward – and very prone to error – because of the different scaling of minutes in the hour, hours in the day and days in the month or year. The MUMPS language is, however, well adapted to handle these scaling differences and the programs that we describe here have been used, with benefit, for a variety of purposes by the staff of this department.

### References

1. Sherertz, D., *ANS MUMPS Programmers' Reference Manual 1983* (MUMPS Users' Group, College Park, 1983), 143.

2. Walters, R. F., Bowie, J. and Wilcox, J. C., *MUMPS Primer*, Revised (MUMPS Users' Group, College Park, 1982), 13.

3. *MIIS Reference Manual*, Version S.MIIS, R-II-3.1 (Medical Information Technology, Inc., Cambridge, 1983), 1.

4. Jablonsky, G., Leung, F. Y. and Henderson, A. R., *Clinical Chemistry*, **31** (1985), 1621.

5. McKenzie, F. N., Moses, G. C. and Henderson, A. R., *Clinical Chemistry*, **31** (1985), 822.

### Appendix

(1) Calculation of time from the S counter of $HOROLOG and displaying it in 24-hour format:
S T=$P ($H, ", ", 2) W T/3600, T#3600/60

(2) Calculation of date (after the year 1900) from the D counter of $HOROLOG and displaying it in DD MM YY format:
S H=$P ($H, ", ",)+1 S YEAR=(H\1461)*4 +1841+H#1461\365
S DAY=H#1461#365 S MONTH=1 I (H#1461) S DAY=366, YEAR=YEAR-1
F I=31, YEAR#4=0+28,31,30,31,30,31,31,30,31,30,31 Q:DAY' >I S DAY=DAY-I, MONTH= MONTH+1
W DAY\10, DAY#10," ", MO\10,MO#10," ", $E(YEAR, 3, 4).

(3) MIIS-specific functions used in the listed programs:

$T — number of elapsed seconds since the first second on 1 January 1976.

$DR — accepts an external date and returns the date expressed as number of days elapsed since 1 March 1800 (reference day).
e.g. C $DR [I, "O"] where I is input (date) and O is output in days since reference day.

$DT — accepts a date expressed as days since 1 March 1800 and returns that date in predefined format.
e.g. C $DT [I, "O", "F"] where I is input (days since reference day) and O is output in predefined format (defined by F).

%TR@I — accepts a specified time and converts it to elapsed time since midnight of the current day.
e.g. C %TR@I [I, "O"] where I is input (time) and O is output (elapsed seconds since midnight).

